# OPTIMAL SELECTION OF BASE MATERIALS FOR ACCURATE DUAL-ENERGY COMPUTED TOMOGRAPHY: COMPARISON BETWEEN THE ALVAREZ–MACOVSKI METHOD AND DIRA

**DOI:** 10.1093/rpd/ncab097

**Published:** 2021-07-08

**Authors:** Maria Magnusson, Gudrun Alm Carlsson, Michael Sandborg, Åsa Carlsson Tedgren, Alexandr Malusek

**Affiliations:** Department of Electrical Engineering; Linköping University, SE-581 83, Linköping, Sweden; Department of Health, Medicine and Caring Sciences; Linköping University, SE-581 83, Linköping, Sweden; Center for Medical Image Science and Visualization (CMIV); Linköping University, SE-581 85, Linköping, Sweden; Department of Health, Medicine and Caring Sciences; Linköping University, SE-581 83, Linköping, Sweden; Center for Medical Image Science and Visualization (CMIV); Linköping University, SE-581 85, Linköping, Sweden; Department of Health, Medicine and Caring Sciences; Linköping University, SE-581 83, Linköping, Sweden; Center for Medical Image Science and Visualization (CMIV); Linköping University, SE-581 85, Linköping, Sweden; Department of Health, Medicine and Caring Sciences; Linköping University, SE-581 83, Linköping, Sweden; Center for Medical Image Science and Visualization (CMIV); Linköping University, SE-581 85, Linköping, Sweden; Department of Medical Radiation Physics and Nuclear Medicine; Karolinska University Hospital, SE-171 77, Stockholm, Sweden; Department of Health, Medicine and Caring Sciences; Linköping University, SE-581 83, Linköping, Sweden; Center for Medical Image Science and Visualization (CMIV); Linköping University, SE-581 85, Linköping, Sweden

## Abstract

The choice of the material base to which the material decomposition is performed in dual-energy computed tomography may affect the quality of reconstructed images. The aim of this work is to investigate how the commonly used bases (water, bone), (water, iodine) and (photoelectric effect, Compton scattering) affect the reconstructed linear attenuation coefficient in the case of the Alvarez–Macovski method. The performance of this method is also compared with the performance of the Dual-energy Iterative Reconstruction Algorithm (DIRA). In both cases, the study is performed using simulations. The results show that the Alvarez–Macovski method produced artefacts when iodine was present in the phantom together with human tissues since this method can only work with one doublet. It was shown that these artefacts could be avoided with DIRA using the (water, bone) doublet for tissues and the (water, iodine) doublet for the iodine solution.

## 1 INTRODUCTION

The Alvarez–Macovski method (AM)^(1)^ and the Dual-energy Iterative Reconstruction Algorithm (DIRA)^(2)^ are image reconstruction algorithms in dual-energy computed tomography (DECT) using a mathematical decomposition of the linear attenuation coefficient (LAC) into energy-dependent basis functions. Both algorithms use energy spectra of photons emitted from the x-ray tube, and both algorithms can produce virtual monoenergetic images at any energy.

The choice of the bases affects the accuracy of the reconstructed LAC and the accuracy of the corresponding weighting coefficients of individual bases. In the medical diagnostics energy range of 20–150 keV, the LAC can be written as a sum of the photoelectric absorption, $\mu _p(E)$, incoherent scattering, $\mu _{inc}(E)$ and coherent scattering, $\mu _{coh}(E)$ components

(1) \begin{align*}& \mu(E) = \mu_p(E) + \mu_{inc}(E) + \mu_{coh}(E). \end{align*}

The incoherent scattering macroscopic cross-section $\mu _{inc}(E)$ takes into account the binding energies of the atomic electrons. It is approximately the same as Compton scattering, which is valid for scattering of a photon against a free electron at rest and is given by the Klein–Nishina cross-section. For soft tissues, the photoelectric effect dominates at energies <20–30 keV, whereas the incoherent scattering dominates at higher energies. For water, coherent scattering contributes about 10% to the total LAC at 20–40 keV. Its role is less important at other energies. Values of $\mu _p(E)$, $\mu _{inc}(E)$ and $\mu _{coh}(E)$ can be obtained from databases like XCOM^(3)^ or EPDL^(4)^, which store cross-sections evaluated from both theoretical calculations and experimental measurements.

In DECT, the linear attenuation coefficient can be decomposed as

(2) \begin{align*}& \mu(E) = w_1 \cdot \mu_1(E) + w_2 \cdot \mu_2(E), \end{align*}

where $w_1$ and $w_2$ are weight coefficients, and $\mu _1(E)$ and $\mu _2(E)$ are the LACs for the two base functions, also called a base doublet.

The AM method performs material decomposition according to equation (2) in the projection domain^(1,5)^. Commonly used base functions are the photo-Compton doublet (PC) used, for instance, by the spectral detector computed tomography (CT) scanner IQon (Philips Healthcare), the water-bone doublet (WB)^(5)^ and the water-iodine doublet (WI)^(6)^ used by General Electric’s DECT scanners. The PC doublet approximates the energy dependence of the photoelectric effect as $\mu _p(E)\sim E^{-3}$ though some other authors also used $\mu _p(E)\sim E^{-2.8}$.^(7)^ The energy dependence of the Compton scattering is given by the Klein–Nishina cross-section. The WB and WI doublets are derived from tabulated data of cross-sections. The AM method uses the calculated weight coefficients $w_1$ and $w_2$ for the computation of monoenergetic images at any energy. This way, beam hardening can be eliminated. A review of the performance and practical applications of the AM method can be found in.^(5)^

DIRA performs the material decomposition in the image domain. Calculated forward projections together with the original projections are used to update the reconstructed image. The image is then decomposed to mass fractions of base materials. It is possible to use different base material doublets at different spatial positions in the image, for instance, for different organs. Alternatively, a three-material decomposition can be used. In this case, the mass density of the mixture is not a free parameter but is calculated from the assumption about the preservation of molar volumes. DIRA does not require geometrically consistent projections and has been extended to the 3D helical geometry.^(8)^

Of interest is the minimum number of basis functions that can reliably represent the LAC of biological tissues in the medical CT energy range 20–150 keV. This problem is known as the intrinsic dimensionality of the cross-section data. Williamson *et al*.^(9)^ investigated biological tissues with effective atomic numbers $Z = 2, \ldots , 20$ in the energy range 20–1000 keV. For determination of individual (mass macroscopic) cross-sections, $\mu _p(E)/\rho $ and $\mu _{inc}(E)/\rho $, where $\rho $ is the mass density, they recommended a water-polystyrene doublet for $Z = 1, \ldots , 8$ and a water-calcium chloride solution for $Z = 8, \ldots , 20$. On the other hand, they claimed that only one base material doublet is sufficient to represent LAC values. Bornefalk^(10)^ applied the Principal Component Analysis to LACs affected by uncertainties for $Z = 1, \ldots , 20$ in the energy range 25–120 keV and discovered an intrinsic dimensionality of 3–4. Alvarez claimed^(11)^ that two base materials are sufficient to represent the attenuation coefficients of biological tissues. However, if a contrast agent with a high atomic number is present, then three or more base materials are needed.

The conflicting results on the dimensionality of the cross-section data raise questions on the cause of this discrepancy and how the choice of the material base affects the material decomposition. This work aims to investigate the problem (1) in the ideal case of a direct decomposition of the LAC to base functions and (2) in the case when the material decomposition is performed by the AM and DIRA reconstruction algorithms on simulated projection data. Such data eliminate machine and quantum noise-related artefacts.

## 2 METHODS

### 2.1 Visual investigation of the dimensionality of relevant materials

Material decomposition to the WB doublet was performed for elements with $Z=1,\ldots ,20$, water, lipid, protein, adipose, muscle, compact bone, femora spongiosa, Ti, Zn, I, Ba and Ce. Reasons for choosing these materials were as follows. Most of the elements comprising human tissues have $Z \le 20$. Soft tissues consist mainly of water, lipids and proteins. Bones consist of compact bone and spongiosa. Ti may be used for implants, Zn can be found in small amounts in the prostate and I, Ba and Ce can be used as contrast agents. True mass attenuation coefficients, $\mu _{\mathrm{m,tab}}$, for these materials were either taken directly from the EPDL97 library^(12)^ or derived from elemental compositions taken from.^(2)^ Corresponding true linear attenuation coefficients were obtained as $\mu _{\mathrm{tab}} = \rho \mu _{\mathrm{m,tab}}$, where $\rho $ is the mass density of the material. The coefficients $w_1$ and $w_2$ in equation (2) are linearly proportional to the density of the decomposed material. Consequently, the ratio $\mu / \mu _{\mathrm{tab}}$, which was used to visually assess the approximation quality, does not depend on the density $\rho $. To evaluate the ratio, the density was set to $\rho = 1 $g cm$^{-3}$ and the weight coefficients $w_1$ and $w_2$ were obtained by solving an equation system consisting of equation (2) at the energy of 50 and 88 keV.

**Figure 1:**
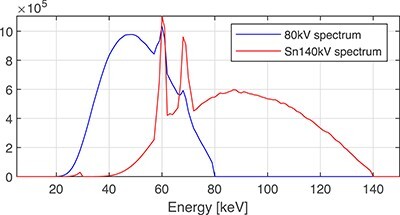
Non-normalised energy distributions of photons in X-ray spectra.

### 2.2 X-ray spectra and phantoms

Photon energy spectra for x-ray tube voltages of 80 and 140 kV were used (Figure 1). The latter spectrum was filtered with an additional tin filter.

**Figure 2:**
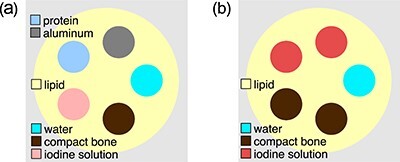
Schematic drawing of the phantom without (a) and with (b) the iodine-water solution. The lipid cylinders with the diameter of 316 mm contained 5 rod inserts with the diameter of 70 mm containing different materials.

Mathematical models of two cylindrical phantoms filled with lipid and containing five rod inserts were used (Figure 2). Rod inserts of the first phantom consisted of water, protein, compact bone, femora spongiosa and aluminium. Rod inserts of the second phantom consisted of water, compact bone and iodine-water solution. The material compositions are shown in Table 1.

**Table 1:** Elemental composition in mass fractions (in %) and corresponding density (in g/cm$^3$) for phantom materials. Compact bone was also used in the WB doublet.

Material	Mass fraction	Density
Lipid	H 11.8, C 77.3, O 10.9	0.92
Protein	H 6.6, C 53.4, N 17.0,	1.35
	O 22.0, S 1.0	
Water	H 11.2, O 88.8	1.00
Compact	H 3.6, C 15.9, N 4.2,	1.92
bone	O 44.8, Na 0.3, Mg 0.2,	
	P 9.4, S 0.3, Ca 21.3	
Femora	H 9.4, C 38.5, N 2.2,	1.124
marrow	O 43, Na 0.2, P 2.2,	
	S 0.3, Cl 0.1, Ca 4.1	
Iodine	I 6, H 10.5184,	1.05024
solution	O 83.4816	

### 2.3 Set-up for DIRA

Projections of the phantom were calculated with Drasim^(13)^ in a fan beam geometry described in^(2)^ and rebinned to parallel projections; this approach was used for the AM too. In the case of DIRA, only the phantom with iodine was processed.

**Figure 3:**
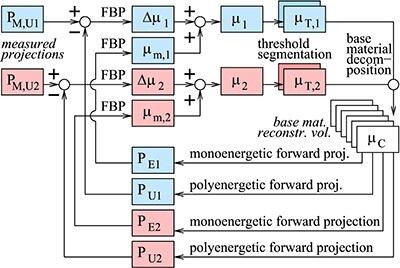
The DIRA.

A short description of the current DIRA algorithm (Figure 3) follows.

1. The simulated *measured projections*  $P_{M,U1}$ and $P_{M,U2}$ for 80 and 140 kV were reconstructed with filtered backprojection (FBP) to $\mu _1$ and $\mu _2$, respectively. The initial reconstruction (iteration 0) was preceded by a conventional water beam-hardening correction.
2. A *threshold segmentation* to $\mu _{T,1}$ and $\mu _{T,2}$ was used to separate regions with iodine from regions with bone and soft tissues. To separate iodine from soft tissue, a threshold at $T=30\ \mathrm{m}^{-1}$ for $\mu _1$ ($E_1 = 50$ keV) was used. To distinguish between iodine and bone, $k=\mu _1/\mu _2$ was calculated. If $k>2.4$, iodine solution was supposed. According to Table 2, compact bone has $k=79.2/38.9=2.04$ and iodine solution has $k=100.0/34.7=2.88$, which justifies the choice of a threshold at 2.4.
3. A *base material decomposition* gave $\mu _C$. In the iodine regions, two-material decomposition to the (iodine, water) doublet was used. In the bone and soft tissue regions, the (compact bone, water) doublet was used.
4. *Monoenergetic forward projections* at $E_1 = 50$ keV and $E_2 = 88$ keV were generated and reconstructed to $\mu _{m,1}$ and $\mu _{m,2}$.
5. *Polyenergetic forward projections* were generated and compared with the simulated measured projections. Reconstruction by FBP gave updates $\Delta \mu _1$ and $\Delta \mu _2$. Addition to $\mu _{m,1}$ and $\mu _{m,2}$ gave $\mu _1$ and $\mu _2$ for the next iteration.

The loop was iterated 16 times but converged earlier. The final $\mu _1$ is the reconstruction for 50 keV, and the final $\mu _2$ is the reconstruction for 88 keV. The reconstruction at additional third energy (30 keV) was easily obtained by combining base materials in $\mu _C$ (at 30 keV).

### 2.4 Set-up for AM algorithm

Projections were calculated as in the previous section. The AM algorithm was implemented according to Ying *et al*;^(14)^ the PC, WB and WI base material doublets were used. The AM reconstruction resulted in two images showing the base material weight coefficients $w_1$ and $w_2$. Averages of $w_1$ and $w_2$ were taken in regions of interest (ROIs) inside the rod inserts and the water cylinder. From these values, LACs as functions of energy in the range 20–150 keV were obtained using Equation (2). Also, three monoenergetic images for 30, 50 and 88 keV were calculated.

## 3 RESULTS

**Figure 4:**
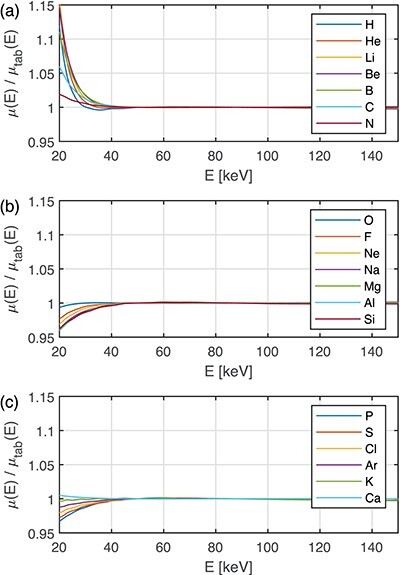
The LAC approximated by the WB doublet relative to the tabulated LAC, $\mu (E)/\mu _{tab}(E)$, as a function of energy for elements with $Z = 1, \ldots , 20$.

**Figure 5:**
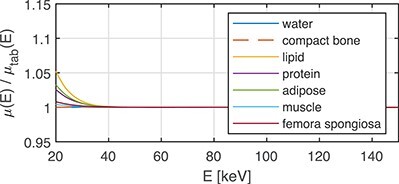
Ratio $\mu (E)/\mu _{tab}(E)$ for common materials, $\mu _{tab}(E)$ is the tabulated LAC and $\mu (E)$ is the LAC approximated with the WB doublet.

The ability of the WB doublet to represent the material of interest is represented by the $\mu (E)/\mu _{tab}(E)$ ratio. This ratio, obtained by direct application of material decomposition via Equation (2), is plotted in Figures 4 and 5 for elements with $Z = 1, \ldots , 20$ and selected common human tissues, respectively.

**Table 2:** LACs (in m$^{-1}$) for the phantom with iodine. Reconstructions by AM with WB, AM with WI, and DIRA. Values deviating >2.4% are marked with an asterisk and values deviating >62% are marked with a bullet. For 88 keV, the discrepancies were small for all methods; the true values were 17.7, 38.9 and 34.7 m$^{-1}$ for water, bone and iodine solution, respectively.

Energy	Material	True	WB	WI	DIRA
	Water	37.6	37.1	37.6	37.6
30 keV	Comp. bone	244.9	245.9	$\bullet $ 91.0	244.4
	Iodine sol.	90.3	$\bullet $ 412.1	90.5	89.4
	Water	22.7	22.6	22.8	22.7
50 keV	Comp. bone	79.2	79.4	$\ast $ 77.3	79.4
	Iodine sol.	100.0	$\ast $ 105.5	100.3	100.3

Note that the elements are well represented for energies above $\approx 35$ keV. For energies in the range 20–35 keV, the discrepancy is larger, especially for H, He, Li, Be, B. However, these substances, except of H, are typically not found in the human body. Cross-sections of compounds containing H are typically dominated by the other elements. In Figure 5, water, bone, lipid, protein, adipose, muscle and femora spongiosa are plotted. For those, the discrepancy in the range 20–35 keV is rather small.

**Figure 6:**
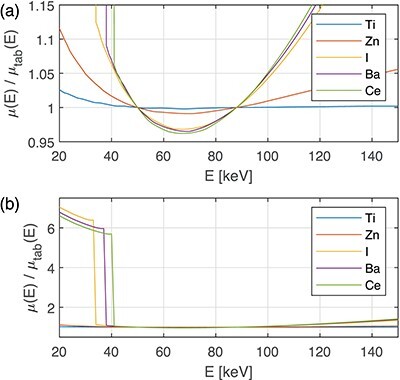
The $\mu (E)/\mu _{tab}(E)$ ratio for some materials of interest in CT, where $\mu _{tab}(E)$ is the tabulated LAC and $\mu (E)$ is the LAC approximated with the WB doublet, plotted using the fine [0.95,1.15] (a) and coarse [0, 8] (b) ranges on the y-axis.

Some other materials of interest for medical CT (Ti, Zn, I, Ba and Ce) are plotted in Figure 6. Note that Ti is represented as good as the human tissues by the WB doublet. Zn is not represented so well, and the contrast agents I, Ba and Ce are represented even worse. The K-edges around 40 keV are an additional complication.

### 3.1 Analysis of reconstructed data for the phantom without an iodine insert

**Figure 7:**
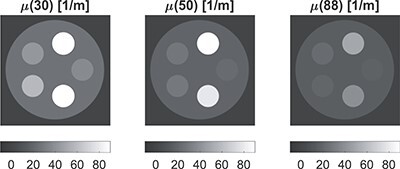
LACs (in $m^{-1}$) for the phantom without iodine reconstructed by AM at 30, 50, and 88 keV with the WB doublet.

**Figure 8:**
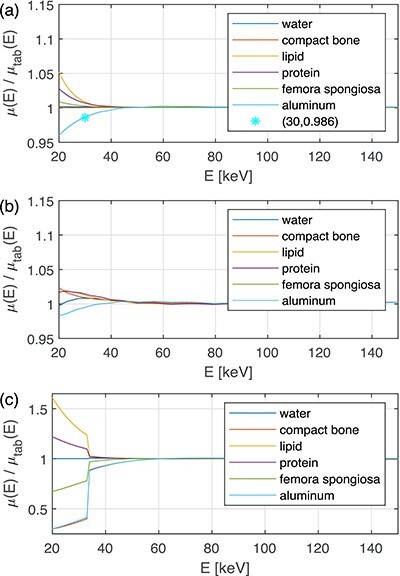
The approximated LAC relative to the tabulated LAC, $\mu (E)/\mu _{tab}(E)$, as a function of energy calculated by the AM for the phantom without iodine and the WB (a), PC (b), and WI (c) doublets. Note the larger range on the y-axis in panel (c).

Figure 7 shows images reconstructed by AM for the WB doublet at 30, 50 and 88 keV. There was a good agreement with tabulated values. The largest difference was for the aluminium rod at 30 keV, with the value $299.6$ m$^{-1}$ measured in a ROI and the tabulated $303.8$ m$^{-1}$, giving a ratio of $299.6/303.8\approx 0.986$. This value agrees with aluminium in Figure 8 (top).

**Figure 9:**
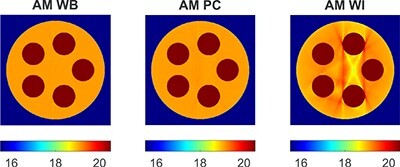
LACs (in m$^{-1}$) reconstructed by AM at 50 keV for the WB, PC, and WI doublets and the phantom without iodine.

Figure 8 shows the $\mu (E)/\mu _{tab}(E)$ ratio obtained by AM for the WB, PC, and WI bases and the phantom without iodine. The WB and PC bases approximated the LAC well for $E>40$ keV. In the range 20–35 keV, the relative difference between the LACs was larger, but it was still <5%. In the case of WI, the K edge of iodine at 33.2 keV caused large discrepancies between the LACs in the range 20–40 keV. These discrepancies lead to a notable beam hardening artefact between the compact bone and aluminium inserts at 50 keV (Figure 9).

Reconstructed images of the phantom without iodine displayed at a reduced LAC window are shown in Figure 9. Contrary to the WB and PC doublets, the WI doublet produced a clearly visible beam hardening artefact. Experiments with DIRA and the WB doublet (not shown here) were in line with AM with WB.

**Figure 10:**
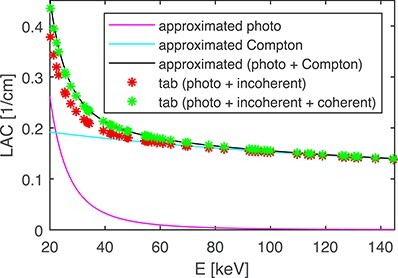
Reconstructed approximated LAC and tabulated LAC for the lipid rod insert in the phantom without iodine as functions of photon energy. The approximated (photo+Compton) LAC agreed well with the tabulated (photo+incoherent+coherent) LAC.

The PC doublet did not provide the correct fractions of the photoelectric and Compton LACs. Nevertheless, Figure 10 shows that it agreed with the tabulated (photo + incoherent + coherent) LAC when both components were added together. This shows that the PC doublet with the $E^{-3}$ dependence can be used in the AM for beam hardening removal but not for the determination of true photoelectric and Compton fractions.

### 3.2 Analysis of reconstructed data for the phantom with an iodine insert

**Figure 11:**
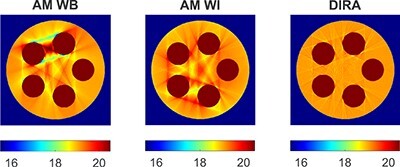
LACs (in m$^{-1}$) for the phantom with iodine reconstructed at 50 keV by AM for the WB and WI doublets and by DIRA.

Images of the phantom with an iodine insert were reconstructed by DIRA and by AM for the WB and WI doublets at 30, 50 and 88 keV. Reconstructed images displayed at a reduced LAC window are shown in Figure 11. Both AM methods produced clearly visible beam hardening artefacts. DIRA reconstructed the phantom without such artefacts. More LAC values for 30 and 50 keV are given in Table 2, where deviating values are highlighted. For 88 keV, the discrepancies were small for all methods; the true values were 17.7, 38.9 and 34.7 m$^{-1}$ for water, bone and iodine solution, respectively.

## 4 DISCUSSION

As mentioned in the introduction section, photoelectric effect, incoherent and coherent scattering contribute to the LAC in the energy range 20–150 keV. The photoelectric effect depends on the atomic number $Z$ as $Z^n$, where $n$ ranges from 4 to 5. Compton scattering (as given by the Klein–Nishina cross-section for scattering against free electrons at rest) used here for incoherent scattering, and coherent scattering are proportional to $Z$ and $Z^2$, respectively. Moreover, the energy dependence of the coherent scattering cross-section is approximately proportional to $E^{-1}$,^(15)^ which emphasises its increasing importance at low photon energies. Thus, the largest discrepancies in the approximation of the LAC are expected at low energies when a combination of both low- and high-$Z$ materials is used. This was observed for (1) the WB base approximating elements with $Z=1,\ldots ,5$ (Figure 4) in the 20–25 keV region, (2) the WB base approximating high $Z$ materials like Zn in the energy range 20–30 keV (Figure 6), (3) high-Z contrast agents like I, Ba and Ce below the K-edge energy (Figure 6), and (4) the WI base approximating the materials in the phantom without iodine (Figure 8). Of special interest is that the deviation was very large when contrast agents were involved, either as a phantom material or as part of the WI base (Figures 6 and 8c). This observation is in line with Alvarez’s statement^(11)^ that two base materials are not sufficient in this case.

The material decomposition in the AM method to the PC base neglects the coherent scattering contribution, but, on the other hand, it uses the Klein–Nishina cross-section, which overestimates the incoherent scattering contribution at low energies, and thus, to a certain degree, compensates for the neglected coherent scattering. This approximation can lead to inaccurate fractions of the photoelectric effect and Compton scattering components. Nevertheless, the sum of both contributions can be biased much less. In our experiments, this behaviour can be seen in Figure 10. A compensation via the energy dependence of the photoelectric effect as $\mu _p(E)\sim E^{-2.8}$ suggested by^(7)^ gave much worse results than $\mu _p(E)\sim E^{-3}$ (results are not presented here).

Figure 4 shows that it may be difficult to reach the dimensionality of 3–4 stated by Bornefalk^(10)^ at the energy range of 35–150 keV for elements with $Z = 1, \ldots , 20$. In this energy range, the dimensionality of 2 is more likely, i.e. one doublet can predict the LAC values of the elements. All the major differences between the LACs of elements are in the energy range 20–35 keV. The relative numbers of photons in the low-energy part of the low- and high-energy X-ray spectra (Figure 1) are small ($<7.9\%$ and $<0.15\%$, respectively) in the range 20–35 keV, (Figure 1). Moreover, in clinical applications, most of these photons are absorbed by adult patient bodies. The situation may be different for children and spectral CT, where low-energy photons may pass the small bodies and contribute to the low-energy channel at the 20–40 keV.

Figure 4 showed how well elements with Z = 1,...20 can be approximated with the WB doublet in the energy range 20–150 keV. A similar investigation was performed by Williamson *et al*.^(9)^ (Fig. 2a) for the polystyrene-calcium chloride solution (PCCS) doublet. The performance of PCCS was better than our WB (Figure 4) in the lower energies for many $Z$ elements. For example, at 20 keV and nitrogen, the relative difference was 2% for WB, but only 1% for PCCS. For energies $>40$ keV, both WB and PCCS gave very small relative differences, <0.1%. On the other hand, for calcium and energies $>110$ keV, the relative difference was $\approx 2\,\%$ for PCCS but <0.1% for WB. The double doublet suggested by^(9)^ did not improve the result for Ca.

The presented work used computer simulations to eliminate machine and quantum noise-related artefacts. In practical applications, however, these will also affect the results, especially for low tube loads, and the quantum noise will decrease the precision of the base material weights. The potentially different sensitivities to the noise of AM and DIRA and the methods for the noise reduction in both algorithms are subjects for future research.

## 5 CONCLUSION

The PC and WB doublets accurately approximated the LAC values for human tissues and elements with $Z = 1, \ldots , 20$, in the 20–150 keV range, though there was a small ($<5\,\%$) discrepancy in the 20–35 keV range. The WI doublet did not represent the tissues as well as PC and WB; the largest discrepancies ($>50\,\%$ in some cases) were in the 20–40 keV range.

LACs reconstructed with the AM and DIRA followed this trend. AM produced artefacts when iodine was present in the phantom together with human tissues, since AM can only work with one doublet. It was shown that these artefacts could be avoided with DIRA using the WB doublet for bone and soft tissues and the WI doublet for the iodine solution.
